# IPHM: Incremental periodic high-utility mining algorithm in dynamic and evolving data environments

**DOI:** 10.1016/j.heliyon.2024.e37761

**Published:** 2024-09-12

**Authors:** Huiwu Huang, Shixi Chen, Jiahui Chen

**Affiliations:** Guangdong University of Technology, School of Computer Science and Technology, Guangzhou, 510006, China

**Keywords:** High-utility itemset, Incremental mining, Pattern mining, Periodic itemset

## Abstract

Periodic high-utility itemset (PHUI) mining can extend beyond the conventional approach of high-utility itemset mining by uncovering recurring customer purchase behaviors common in real-life scenarios (e.g., buying apples and oranges every three days or weekly). Such behaviors, particularly in market basket databases, signify stable patterns that ensure long-term profitability. Existing PHUI mining algorithms assume a static database and incur significant costs when handling incremental databases, as each batch of new transactions necessitates reprocessing the entire dataset. To overcome this challenge, we introduce the Incremental Periodic High-Utility Itemset Miner (IPHM), a method for efficiently extracting periodic high-utility itemsets in incremental database environments. We propose an innovative incremental utility-list structure tailored for incremental database scenarios. Effective pruning strategies are employed to expedite the construction and update of incremental utility-lists and to discard unpromising candidates. As demonstrated by the experimental results, the algorithm is efficacious and efficient, highlighting its practical applicability in dynamic data environments. The algorithm shows a remarkable ability to quickly adapt to database changes, making it highly suitable for applications in market basket analysis where frequent updates are common.

## 1 Introduction

High utility itemset mining (HUIM) [1] is a significant research area within data mining that has recently attracted considerable research interest. Inspired by traditional mining methods, such as Frequent Itemset Mining (FIM) [2], HUIM extends the concept by considering transaction scenarios where items appear multiple times and are assigned individual utility values. An itemset is considered a High-Utility Itemset (HUI) if its utility value meets or surpasses a predefined minimum utility threshold. HUIM aims to identify every itemset with high utility within a transaction database. HUIM has practical uses in diverse fields, such as targeted product recommendations in e-commerce, financial risk assessment, and even patient treatment analysis in healthcare [1]. HUIM has led to the development of specialized data mining tasks such as high average-utility itemset analysis [3], [4], [5] and periodic high-utility itemset [6], [7], [8] mining, among others. Recently, new methods have been developed for extracting High-Utility Itemsets (HUIs) in both static databases through traditional methods [9], [10], [11] and dynamic databases through incremental methods [12], [13], [14] with transaction insertions. However, these methods, primarily designed for HUI mining tasks, face a notable challenge: the HUI sets identified by traditional HUIM can be excessively large, complicating the task for analysts to extract actionable insights efficiently. Furthermore, traditional algorithms in HUIM are not adept at identifying repetitive purchasing behaviors, a scenario frequently encountered in real-world settings. For instance, in a retail store, certain combinations of products may be consistently purchased together daily or weekly. Accurately capturing this information can significantly enhance understanding of customer purchasing behaviors, guiding procurement and sales staff. For example, procurement staff can make informed decisions on restocking these product combinations, while sales staff can design targeted promotional activities around them.

Responding to this challenge, Fournier-Viger et al. [6] introduced a method integrating periodicity analysis into HUIM, aimed at mining a type of HUIs known as periodic high-utility itemsets(PHUIs). These PHUIs offer richer information, enhancing the utility and relevance of the mined itemsets. Drawing inspiration from Periodic Frequent Pattern (PFP) analysis, PHUI effectively uncovers patterns in transaction databases that adhere to periodic constraints. However, Periodic Frequent Patterns focus solely on the frequency and intervals of pattern occurrence, lacking the capacity to identify high-utility periodic patterns. Consequently, the proposed PHUIs encompass patterns' periodic and utility measures, ensuring that profitable periodic patterns are not overlooked. However, the approach for mining PHUIs assumes that the transaction database is static and does not account for dynamic databases. To my knowledge, no method currently exists capable of mining and maintaining PHUIs in an incremental database, signifying a gap in the existing research and a potential area for future development. In real-world scenarios, particularly in sectors like retail, new transactions are continuously occurring, leading to dynamic database changes. Traditional methods tailored for static databases fail to accommodate this dynamism. Relying on these static approaches in such evolving environments could lead to inefficient resource use and missed opportunities for actionable insights. Developing techniques for mining and maintaining PHUIs in such incremental databases is crucial. To address these challenges, we propose a novel algorithm, IPHM (Incremental Periodic High-Utility Itemset Miner), for the incremental mining of PHUIs. The IPHM algorithm is designed to efficiently adapt to dynamically evolving databases, specifically by updating the PHUIs as new transactions are added, thereby offering a more practical and real-time solution for mining in dynamic retail environments and similar scenarios. Specifically, this work advances research through the following contributions:

1) We introduce incremental utility lists that can exploit early pruning and propose a refactoring method for item utility tables when transactions are inserted to keep utility lists valid. Our approach eliminates the utility of traversing the entire incremental database again for each update and greatly improves efficiency by reducing redundant computations.
2) We proposed IPHM, a novel algorithm for efficient PHUI mining in incremental databases. It employs a specialized incremental utility-list structure to achieve this. Designed for high performance, IPHM achieves effective utility-list construction or reconstruction for individual items with a single pass through the original database and inserted transactions.
3) To assess IPHM's performance, we conducted extensive experiments on databases with diverse characteristics. In these experiments, we compared the IPHM with the latest algorithms for mining PHUIs in static databases. This comparative analysis provided insights into the efficiency, scalability, and effectiveness of IPHM, particularly in dynamic and evolving data environments.

The rest of this paper is structured as follows: Section 2 provides problem definitions and establishes essential preliminaries. Section 3 presents the related work, providing context and background. Section 4 provides an in-depth examination of the IPHM algorithm, outlining its core components and how they function together. Section 5 is devoted to experiments and result analysis, where we present compelling evidence for IPHM's effectiveness and efficiency. Finally, Section 6 concludes with a recap of the research contributions and outlines potential directions for further extending this work.

## 2 Related work

This section reviews concepts and methodologies about high-utility itemset mining, periodic frequent pattern mining, and periodic-based high-utility itemset mining.

### 2.1 High-utility itemset mining

HUIM extends Frequent Itemset Mining (FIM) [2] by focusing on discovering high-utility itemsets in transaction databases. These itemsets represent groups of items that generate high profit or utility, rather than just frequent appearances. This approach considers each item's non-binary purchase quantities and individual weights (e.g., unit profit), making the task more complex than FIM. In real-life situations, such as retail store management, HUIM is used to understand profitable sets of items customers buy, aiding in strategic marketing decisions.

Unlike the support measure's inherent downward closure property, the utility measure in HUIM lacks this characteristic. To overcome the challenge posed by the lack of downward closure in HUIM's utility measure, Liu et al. [15] introduced Transaction-Weighted Utility (TWU) as an upper bound estimate for itemset's utility. The TWU measure possesses anti-monotonicity, a crucial feature that facilitates efficient reduction of the search space in HUIM. Methods [10], [15] employing the TWU measure can effectively narrow down the search space without risking omitting HUIs. This advancement has significantly streamlined the process of identifying HUIs, addressing one of the primary challenges in the field of HUIM. However, a limitation arises with using TWU in HUIM, as TWU represents a loose upper bound of an itemset's utility. This characteristic of TWU leads to algorithms considering numerous potential itemsets for mining HUIs, which may not substantially improve efficiency. The broad upper bound set by TWU results in exploring many itemsets that, while not missing any potential HUIs, also include numerous non-essential candidates. Consequently, this can lead to only marginal gains in efficiency, thus presenting a challenge in optimizing the HUIM process.

Many high-utility pattern mining algorithms [10], [15], [16], [17] incorporate the pruning strategy pioneered by Liu et al. [15], enabling effective search space reduction. These algorithms typically follow a two-phase approach. A major challenge with these algorithms is assessing many candidate itemsets to identify the final high-utility itemsets. This inefficiency arises from their reliance on a loose upper bound for TWU. To overcome the issue of a substantial number of candidate itemsets in HUIM, several algorithms [11], [18], [19], [20], [21], [22] have been developed that implement one-phase mining of high-utility patterns. These methods utilize a structure known as utility-list, combined with effective pruning strategies. Experimental results have demonstrated that one-phase algorithms are more efficient than their two-phase counterparts.

Then, a novel algorithm named R-Miner [23] is introduced, which utilizes two innovative data structures, the residue map and master map. This approach significantly enhances mining efficiency, achieving up to twice the performance of existing list-based algorithms.

Finally, it is worth noting that the tree-based method named EFIM [24] is another effective approach for mining high-utility itemsets. This method constructs a tree structure to store utility information, enabling efficient mining of high-utility itemsets. A novel method named Hamm [25] employs a TV(prefix Tree and utility Vector) structure, which combines a prefix tree and a utility vector to efficiently store item and utility information in transaction databases, thereby supporting the efficient mining of high-utility itemsets.

### 2.2 Periodic-frequent pattern mining

In response to a prevalent issue in Frequent Itemset Mining (FIM) algorithms, where an excessive number of frequent patterns are mined, many of which may not be interesting to users, algorithms for mining Periodic Frequent Patterns (PFP) have been proposed. Periodic-frequent pattern mining (PFPM) [26], [27], [28] is also the extension of FIM. These algorithms aim to refine the mining process by focusing on patterns that appear frequently and do so periodically.

PFPM is widely applied across diverse fields, including retail analysis, biomedical research, network security, telecommunications, supply chain management, and energy consumption analysis. It offers valuable insights by identifying recurrent patterns in data.

### 2.3 Periodic-based high-utility itemset mining

Traditional HUIM algorithms often fall short of capturing recurring customer purchase behavior. To address this limitation, researchers introduced the concept of periodic high-utility itemset mining (PHUIM). This approach aims to discover itemsets exhibiting high utility and regular purchase patterns, which are common in many real-life scenarios, such as regular purchases in retail stores. Introducing PHUIM helps identify profitable and recurring patterns, providing more profound insights into customer buying habits.

The PHM [6] algorithm introduces the task of mining periodic high-utility itemsets, highlighting the limitation of traditional HUIM algorithms in identifying recurring customer purchase behaviors. It also incorporates average periodicity as a novel measure, offering a more nuanced assessment of a pattern's periodic nature. The PHMN+ [8] algorithm extends traditional periodic high-utility itemset mining by addressing the challenge of handling negative utility items. The PPHUI [7] algorithm was developed to mine partial periodic high-utility itemsets in situations where time intervals are inconsistent and external utilities vary over time.

Fournier-Viger et al. [29] discussed potential research opportunities for discovering high-utility itemsets with periodicity or irregularity in transaction databases.

### 2.4 Comprehensive comparison

We compare our work with related works, as shown in Table 1, highlighting their different characteristics. Our algorithm demonstrates high efficiency primarily in incremental dataset environments.

**Table 1:** Comprehensive comparison of related work on algorithms.

Algorithms	Periodic	High-utility	Incremental	Time consume	Memory Usage
two-phase [15]	×	✓	×	slow (tree-based)	high
FHM [11]	×	✓	×	partly fast (list-based)	partly high
EFIM [24]	×	✓	×	partly fast (projection-based)	partly low
R-Miner [23]	×	✓	×	fast (list-based)	low
PHM [6]	✓	✓	×	partly slow (list-based)	partly low
PHMN+ [8]	✓	✓	×	partly fast (list-based)	partly low
PPHUI [7]	✓	✓	×	partly fast (tree-based)	partly high
TSPIN [26]	✓	×	×	partly fast (tree-based)	low
ours	✓	✓	✓	fast (list-based)	partly low

## 3 Preliminaries and problem definition

This section establishes the theoretical background for this work, covering high-utility itemset mining and periodic frequent pattern mining, leading to the formal definition of the problem of incremental periodic high-utility itemset mining.

### 3.1 Basic preliminaries

In this paper, we assume a set of distinct items represented as $I=\{i_{1},i_{2},\ldots ,i_{m}\}$, where *m* denotes the total number of items. Each item $i_{j}$ in the set *I* has an associated positive value, $p(i_{j})$, representing its external utility (e.g., unit profit). This value reflects the item's significance or weight to the user. Then, we assume the itemset $X=\{x_{1},x_{2},\ldots ,x_{k}\}$, comprised of *k* distinct items, is considered an *k*-itemset if it is a subset of the overall item set *I* where |X|=k. Let $D=\{T_{1},T_{2},\ldots ,T_{n}\}$ be a quantitative transaction database that contains *n* transactions. The transaction database *D* contains *n* transactions, where each transaction $T_{c}\subseteq I$ (1≤c≤n), and is uniquely identified by an index *c*, known as the Transaction ID (Tid). Furthermore, each item $i_{j}$ within the transaction $T_{c}$ is assigned a positive integer value, $q(i_{j},T_{c})$, representing its internal utility (e.g., purchase quantity).

### 3.2 High-utility itemset

Definition 1 **Utility of an item/itemset**

The utility of an item $i_{j}$ in a transaction $T_{c}$, denoted by $u(i_{j},T_{c})$, is calculated as the product of its internal utility ($q(i_{j},T_{c})$) and external utility ($p(i_{j})$) within that transaction. Mathematically, this is expressed as: $u(i_{j},T_{c})=q(i_{j},T_{c})\times p(i_{j})$. The utility of an itemset *X* in a transaction $T_{c}$, denoted by $u(X,T_{c})$, is calculated as the sum of the utilities of individual items within that itemset. In other words, for each item $i_{j}$ belonging to *X*, its utility $u(i_{j},T_{c})$ (as defined previously) is considered. Formally, this is expressed as:

$$u(X,T_{c})=\left\{ \begin{array}{cc} \sum_{i\in X}u(i,T_{c}),\; & \text{if }X\subseteq T_{c}\\ 0,\; & \text{otherwise} \end{array}\right.$$

Furthermore, the utility of an itemset *X* across the entire database is denoted by u(X). It is calculated by summing the utility of *X* in each transaction that contains it. Mathematically, this is represented as: $u(X)=\sum_{T_{c}\in g(X)}u(X,T_{c})$, where g(X) signifies the set of all transactions in the database that contain the itemset *X*.

Example 1 To illustrate, let's consider the original database *D* shown in Table 2 and the associated unit profits of items in Table 3, $u(e,T_{1})=1\times 2=2$, and $u(\{b,d\},T_{2})=1\times 3+2\times 4=11$. The utility of {b,d} is $u(\{b,d\})=u(\{b,d\},T_{2})+u(\{b,d\},T_{5})=11+7=18$.

**Table 2:** Original database *D* and incremental database *N*.

Database	Tid	Transaction	TU
*D*	*T*_1_	(a,1), (c,1), (e,1)	8
	*T*_2_	(b,1), (d,2), (f,1), (g,1)	16
	*T*_3_	(a,1), (d,1), (e,2), (f,2), (g,1)	21
	*T*_4_	(c,3), (e,1), (f,1)	8
	*T*_5_	(a,1), (b,1), (d,1), (e,1), (g,2)	18

*N*	*T*_6_	(b,2), (c,1), (e,3), (g,1)	15
	*T*_7_	(a,3), (d,2), (f,2)	29

**Table 3:** Unit profit for items a to g.

Item	a	b	c	d	e	f	g
Unit profit	5	3	1	4	2	3	2

Definition 2 **High-utility itemset**

Giving a user-specified minimum utility threshold named minutil>0. An itemset *X* must satisfy u(X)≥minutil to be considered a high-utility itemset (HUI).

Example 2 For the original database *D* in Table 2, given that minutil=20. The utility of {b,d,g} is $u(\{b,d,g\})=u(\{b,d,g\},T_{2})+u(\{b,d,g\},T_{5})=24$, which is no less than *minutil*. Therefore, we can categorize the itemset {b,d,g} as an HUI.

The primary objective of high-utility itemset mining (HUIM) is the discovery of all HUIs within a dataset. Because utility attributes do not adhere to the downward-closure property, an itemset can be considered a High-Utility Itemset (HUI) even if its subsets are not. Several HUIM algorithms rely on the transaction-weighted utilization (TWU) attribute to satisfy the downward-closure property. However, this approach often leads to overestimated utility values for itemsets. The definition of TWU is as follows.

Definition 3 **Transaction-weighted utilization**

The utility of a given transaction $T_{c}$, represented as $TU(T_{c})$, is quantified by the aggregate of utility values of the items it contains. Mathematically, this is defined as: $TU(T_{c})=\sum_{i\in T_{c}}u(i,T_{c})$. The transaction-weighted utilization (TWU) of an itemset *X* is signified by TWU(X), which means the sum of the utilities of all transactions that contain *X*. TWU is defined as $TWU(X)=\sum_{T_{c}\in g(X)}TU(T_{c})$.

Example 3 For example, consider the original database *D* as outlined in Table 2. The utility of the transaction $T_{1}$ is given by $TU(T_{1})=u(a,T_{1})+u(c,T_{1})+u(e,T_{1})=5+1+2=8$. For the itemset {f,g}, the transaction-weighted utilization (TWU) is calculated as $TWU(\{f,g\})=TU(T_{2})+TU(T_{3})=16+21=37$.

Theorem 1 **Pruning using the TWU**

*Let there be an itemset X, if* TWU(X)<minutil *, then X and all its supersets cannot be high-utility itemsets.*

Proof This theorem is based on the work by Liu et al. [15]. According to their findings, itemsets can be effectively pruned using the TWU value. Specifically, if the TWU of an itemset *X* is less than a given minimum utility threshold (*minutil*), then *X* and all its supersets cannot be high-utility itemsets. This allows for efficient pruning in the search space of potential high-utility itemsets.

To improve efficiency, many two-phase algorithms for HUI extraction leverage the TWU attribute for effective search space pruning [10], [15], [16], [17]. The execution process of these algorithms is mainly divided into two phases: Firstly, they calculate TWU values to identify potential high-utility itemset candidates. Secondly, they scan the database to calculate the actual utility of these candidates, filtering out those with low utility. The TWU upper-bound, while useful, has the significant drawback of being a loose estimate. This overestimation can lead to unnecessary exploration of unpromising itemsets. This loose bound generates many potentially irrelevant candidates, necessitating expensive utility calculations for each. To overcome this challenge, several algorithms adopt a one-phase [11], [18], [24] approach for high-utility itemset mining, providing a more efficient alternative. For example, HUI-Miner [18] employs a utility-list structure to efficiently store and process utility information for each itemset. The utility-list is a simple data structure that facilitates mining by extracting information directly from the database, eliminating the need for repeated scans. A key advantage of utility-lists is that they support efficient join operations, allowing the utility-list of an itemset to be derived directly from the utility-lists of its subsets. Let's define a utility-list formally.

Definition 4 **Utility-list [18]**

Let ≻ denote a total order on items from *I*. The utility-list corresponding to an itemset *X*, defined as ul(X), consists of a set of triples (tid,iutil,rutil), with each triple corresponding to a transaction $T_{tid}$ that includes *X* ($X\subseteq T_{tid}$). Within each tuple of the utility-list: The iutil element represents the direct utility of itemset *X* within the transaction $T_{tid}$, denoted by $u(X,T_{tid})$. The rutil element signifies the remaining utility of items appearing after *X* (according to the total order ≻) within the transaction $T_{tid}$. Mathematically, this is calculated as: $\sum_{i\in T_{tid}\wedge (\forall x\in X)i≻x}u(i,T_{tid})$.

Example 4 Considering the original database *D* and assume a lexicographical ordering of items (represented by ≻). The utility-list for the item {b} is $\left\{(T_{2},3,13),(T_{5},7,10)\right\}$. The utility-list for the itemset {a,e} is $\left\{(T_{1},7,0),(T_{3},9,8),(T_{5},7,4)\right\}$.

To discover HUIs, HUI-Miner [18] performs a single database scan to construct utility-lists for single items. Subsequently, it generates longer itemsets by performing join operations on the utility-lists of subsets. Based on HUI-Miner, the innovation of FHM [11] lies in the proposal of a new strategy based on item co-occurrence analysis, EUCP (Estimated Utility Co-occurrence Pruning), which aims to reduce the number of join operations during the process of mining high-utility itemsets, thereby significantly improving algorithm efficiency. The utility-list structure enables efficient calculation of itemset utilities and facilitates search space pruning, further detailed in the following property and theorem.

Property 1 **Utility calculation via utility-list**

*[18]* *The utility of an itemset X is directly derived by summing the* iutil *values of all tuples within its associated utility-list,* ul(X) *.*

Theorem 2 **Pruning using the sum of iutil and rutil values [18]**

*Let X be an itemset, and let its expanded itemsets be formed by appending an item y such that* y≻i *,* ∀i∈X *. If the following condition holds:*

$$\sum_{\left(tid,iutil,rutil)\in ul(X\right)}(iutil+rutil)<\mathrm{minutil},$$

*then itemset X and all its expansions are guaranteed to be low-utility itemsets.*

Proof This theorem is based on the work by Liu et al. [18]. Specific evidence can be found in the referenced literature.

Although these one-phase high-utility itemset mining algorithms are highly effective, they do not consider the discovery of periodic patterns.

### 3.3 Periodic-frequent pattern

The field of traditional data mining has seen the development of numerous algorithms for discovering periodic-frequent patterns (PFPs) [26], [27], [28] within transaction databases. An itemset that appears periodically and frequently within a database is referred to as a periodic-frequent pattern. Periodic-frequent patterns are particularly interested in certain scenarios, such as web browsing and market sales analysis, where they reveal long-term repetitive habits of users. The concept and definition of PFP are elucidated as follows.

Definition 5 **Periods of an itemset [27]**

Consider a database $D=\{T_{1},T_{2},\ldots ,T_{n}\}$ consisting of *n* transactions, where its transactions are ordered by time, and an itemset *X*. The set of transactions containing *X* is denoted as $g(X)=\{T_{g_{1}},T_{g_{2}},\ldots ,T_{g_{k}}\}$, where $1\leq g_{1}<g_{2}<\ldots <g_{k}\leq n$. If there is no transaction $T_{w}\in g(X)$ such that x<w<y, then two transactions $T_{x}⊇X$ and $T_{y}⊇X$ are considered to be consecutive regarding *X*. The period between two adjacent transactions $T_{x}$ and $T_{y}$ within g(X) is defined as $\text{per}(T_{x},T_{y})=(y-x)$, which denotes the count of transactions between $T_{x}$ and $T_{y}$. Then, the period of the itemset *X* is a list of periods, defined as $\text{ps}(X)=\{g_{1}-g_{0},g_{2}-g_{1},\ldots ,g_{k+1}-g_{k}\}$, where $g_{0}$ (set to 0) represents the position before the first transaction containing *X*, and $g_{k+1}$ (set to n+1) represents the position after the last transaction containing *X*. Thus, ps(X) is expressed as ${⋃}_{1\leq x\leq k+1}(g_{x}-g_{x-1})$.

Example 5 Considering the original database *D* and incremental database *N*. For the itemset {d,f}, the transactions set containing it is $g(\{d,f\})=\{T_{2},T_{3},T_{7}\}$. Thus, the period of the itemset {d,f} is ps({d,f})={2,1,4,1}.

Definition 6 **Periodic-frequent pattern [27]**

Giving a user-specified maximum periodicity threshold named maxPer>0. The itemset *X*'s maximum periodicity is defined as maxper(X)=max⁡(ps(X)). To be considered a periodic-frequent pattern (PFP), an itemset *X* must satisfy maxper(X)≤maxPer.

Theorem 3 **Periodic pruning strategy**

*Let X be an itemset. Its expanded itemsets are formed by appending an item y where* y≻i *,* ∀i∈X *. If* maxper(X) *is bigger than maxPer, itemset X and its extensions fail to meet PFP criteria.*

Proof Assume maxper(X)>maxPer. By definition, maxper(X) is the maximum period of itemset *X*. For any expansion $X^{\prime }=X\cup \{y\}$, $\mathrm{maxper}(X^{\prime })\geq \mathrm{maxper}(X)$. Since maxper(X)>maxPer, it implies $\mathrm{maxper}(X^{\prime })>\mathrm{maxPer}$. Hence, *X* and $X^{\prime }$ do not satisfy the PFP criteria.

### 3.4 Problem statement

Definition 7 **Periodic high-utility itemset**

Giving two user-specified thresholds maxPer>0 and minutil>0, an itemset *X* is considered a periodic high-utility itemset (PHUI) if it satisfies both u(X)≥minutil and maxper(X)≤maxPer.

Problem statement This research tackles incremental periodic high-utility itemset mining (PHUI) in dynamic environments. Here, the incremental database (denoted as *DN*) is formulated by the union of the original database *D* and a set of new, non-empty transactions, represented as *N*, where DN=D∪N. We should continuously extract periodic high-utility itemsets in *DN*. A key parameter in this task is the *minutil*, which is recalculated to reflect changes in the database's total utility. This recalculated minutil threshold is defined as minutil=minutil percentage×u(DN). Along with this adaptive minutil threshold, the mining process also adheres to predefined *maxPer* thresholds.

## 4 The IPHM algorithm

To address the limitations previously identified in handling transaction insertions in databases, this section introduces the IPHM algorithm, which effectively overcomes these challenges. The IPHM algorithm employs a specialized incremental utility-list structure to efficiently discover PHUIs within dynamic databases. Specifically, the IPHM algorithm operates in three stages:

1) This stage involves reading the original database or inserted transactions. Items that meet the maxPer threshold are retained for further processing. This phase initializes a global list for single items' utility-lists and maintains the incremental utility-list for each item that satisfies the maxPer threshold.
2) In this stage, items are sorted based on their TWU values, which determine the global list's order.
3) This final stage executes a recursive search process to mine PHUIs.

### 4.1 Incremental utility-list structure

IPHM is a utility-list based algorithm inspired by PHM [6]. We have innovated the traditional utility-list structure to accommodate the nuances of incremental databases. The modifications made to the utility-list structure are designed to enhance its adaptability and efficiency in dynamically changing data environments. The following definition and properties describe the incremental utility-list structure:

Definition 8 **Order of items**

Considering a set of items, denoted by $I^{\prime }=\{i_{1},i_{2},\ldots ,i_{m}\}$, this work defines an order relation denoted by ≺ on the items within $I^{\prime }$. For any two items $i_{j}$ and $i_{k}$ belonging to $I^{\prime }$, the order is determined by comparing their Transaction Weighted Utility (TWU) values. Specifically, if $TWU(i_{j})<TWU(i_{k})$, then we define $i_{j}≺i_{k}$. Additionally, in the case where the TWU values are identical, the items are then ordered alphabetically.

This ordering prioritizes items with lower TWU values, a strategic approach that facilitates the efficient traversal and processing of the itemset in the mining algorithm. [18]

Example 6 Considering the original database *D* as presented in Table 2, we calculate the TWU value for each item, the results are presented in Table 4. Following the ascending order of TWU values, the items are sorted as c≺b≺f≺a≺d≺e≺g. Additionally, considering both the original database, denoted by *D*, and the incremental transaction set, denoted by *N*, as presented in Table 2, we recalculate the TWU values for each item. The updated results are presented in Table 5. Consequently, the new order of the items becomes c≺b≺e≺g≺f≺a≺d.

**Table 4:** Calculation of TWU values for each item in the original database *D*.

Item	a	b	c	d	e	f	g
TWU	47	34	16	55	55	45	55

**Table 5:** Calculation of TWU values for each item in the original database *D*.

Item	a	b	c	d	e	f	g
TWU	76	49	31	84	70	74	70

Definition 9 **Incremental utility-list structure**

Let ≻ define a total ordering of items from the set *I*. Assume that *TD* is the set of transactions from the original database *D*, and *TN* is the set of transactions from the non-empty transactions *N*, TDN=TD∪TN. The incremental utility-list corresponding to an itemset *X*, defined as iUL(X), comprises two parts of the traditional utility-list, defined as Definition 4: one for storing information related to the itemset *X* in the original database *D*, denoted as *ulD*, and the other for the incremental part *N*, denoted as *ulN*. Formally, iUL(X)=iUL(X).ulD∪iUL(X).ulN. Additionally, the incremental utility-list is annotated with one additional value: maxper(X).

Using this definition, we establish the following theorem for calculating an itemset's utility directly from its incremental utility-list.

Theorem 4 **Sum of iutil values**

*Let there be an itemset X. The utility of X,* u(X) *, is determined by summing the* iutil *values within its incremental utility-list,* iUL(X) *. Thus, an item X is considered a HUI if the sum of its* iutil *values exceeds or meets the minutil threshold.*

Proof Based on Definition 4, we know that the iutil values in the incremental utility-list iUL(X) represent the utility contributions of itemset *X* in their respective transaction $T_{c}$. The total utility of *X*, denoted as u(X), is the sum of these iutil values across all transactions where *X* appears. Mathematically, this can be expressed as: $u(X)=\sum_{e\in iUL(X)}iutil(e)$, where *e* represents an entry in iUL(X) and iutil(e) is the utility of *X* in the corresponding transaction. An itemset *X* is considered a High Utility Itemset (HUI) if u(X)≥minutil, where *minutil* is the minimum utility threshold. Therefore, if the sum of the iutil values of *X* meets or exceeds *minutil*, *X* qualifies as a HUI.

Theorem 5 **Pruning using the increment utility-list**

*Let there be an itemset X. If the sum of* iutil *and* rutil *values within its incremental utility-list,* iUL(X) *, is less than the threshold minutil, then X and its extensions (formed by appending an item y where* y≻i,∀i∈X) *are classified as low-utility.*

Proof Based on the Definition 4 of iutil and rutil, we know that:

iutil(e) represents the utility of itemset *X* in the transaction corresponding to entry *e* in iUL(X). rutil(e) represents the remaining utility of the items in the transaction that can potentially be added to *X* to form extensions.

The sum of iutil and rutil values for an itemset *X* can be expressed as:

$$iU(X)+rU(X)=\sum_{e\in iUL(X)}\left(iutil(e)+rutil(e)\right)$$

where iU(X) is the total utility of *X* and rU(X) is the total remaining utility that can be added to *X*.

If the sum iU(X)+rU(X) is less than the threshold *minutil*, it implies that even by considering the maximum possible utility contributions from extending *X*, the total utility will still be less than *minutil*. Therefore, itemset *X* and any of its extensions $X^{\prime }=X\cup \{y\}$ (where y≻i,∀i∈X) cannot meet the minimum utility threshold *minutil*.

**Figure 1:**
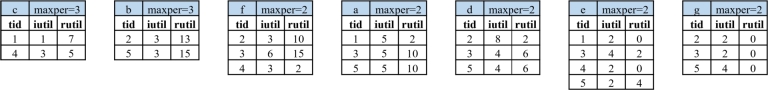
Incremental utility-lists of single items for the original database *D*.

Example 7 Consider the original database *D*, as depicted in Table 2. We first compute each item's TWU values. The results are tabulated in Table 4. After ordering items in ascending TWU sequence, we obtain the sequence c≺b≺f≺a≺d≺e≺g. Subsequently, the incremental utility-lists for single items are derived, as shown in Fig. 1. Let's assume that minutil=25. The total utility for an item *f* is computed by aggregating the iutil values within its utility-list: u(f)=3+6+3=12, which classifies *f* as a low-utility item. However, the sum of iutil and rutil values within iUL(f), which equals 3+6+3+10+15+2=39≥minutil. Therefore, we need to continue to consider the item *f* and its extensions.

### 4.2 The EUCS

Definition 10 **Estimated Utility Co-occurrence Structure [11]**

For any two distinct items $i_{j}$ and $i_{k}\in I^{⁎}$, which is a set of promising single items (i.e., items for which maxper(i)⩽maxPer and TWU(i)⩾minutil), we store the TWU value of the itemset $\left\{i_{j},i_{k}\right\}$ in a triangular matrix. The TWU value of the itemset $\left\{i_{j},i_{k}\right\}$ can be indexed by (j,k). We term this matrix the Estimated Utility Co-occurrence Structure (EUCS).

Theorem 6 **Estimated Utility Co-occurrence Pruning**

*Consider an itemset X containing two distinct items* $i_{j}$ *and* $i_{k}$ *, and the TWU value for the pair can be retrieved from the EUCS using the index* (j,k) *, then if* $TWU(\{i_{j},i_{k}\})<\mathrm{minutil}$ *, neither X nor its extensions qualify as high-utility itemsets.*

Proof This theorem is based on the work by Fournier-Viger et al. [11]. According to their study, the Transaction Weighted Utility (TWU) of an itemset $\left\{i_{j},i_{k}\right\}$ provides an upper bound on the utility of any itemset containing $i_{j}$ and $i_{k}$. If the TWU of $\left\{i_{j},i_{k}\right\}$ is less than the minimum utility threshold *minutil*, then the maximum possible utility of any itemset containing $\left\{i_{j},i_{k}\right\}$ will also be less than *minutil*.

Example 8 If there be a single items set I={a,b,c,d} having four items. The corresponding EUCS is illustrated in Table 6.

**Table 6:** The EUCS.

	*a*	*b*	*c*	*d*
*a*		*TWU*({*a*,*b*})	*TWU*({*a*,*c*})	*TWU*({*a*,*d*})
*b*			*TWU*({*b*,*c*})	*TWU*({*b*,*d*})
*c*				*TWU*({*c*,*d*})
*d*

In the proposed algorithm IPHM, we just construct the EUCS for the single items that pass the periodic pruning strategy shown in Theorem 3. Since the maximum period of each item is determined during the first database traversal, we can strategically reduce the number of items through periodic pruning, reducing the size of the EUCS.

### 4.3 Maintain the order of items/incremental utility-lists

This section details our methods for sorting single items and managing their associated utility-lists.

We employ the proposed incremental utility-list structure to efficiently preserve essential information about single items. The incremental utility-lists are sorted based on the order of the items. However, when the incremental database *N* is integrated, the order of the items may change, leading to modifications in the rutil values of the incremental utility-list.

Property 2 *Although the items order changes, the* iutil *values in the ulD and ulN components of the incremental utility-list remain unchanged. However, the change in item order invalidates previously calculated* rutil *values, as these depend on the original ordering.*

Proof The rutil values are derived from the order of the items. With the insertion of the incremental database *N*, it is necessary to recompute the TWU values for individual items. A new order of items, denoted by ≻, is sorted based on ascending TWU values. As the rutil value represents the aggregate utilities of subsequent items, it must be recalculated after the item reordering. Meanwhile, the iruti values, based on item utility values, are calculated by multiplying the item's quantity by its unit profit. These values do not change with the reordering of items.

**Figure 2:**
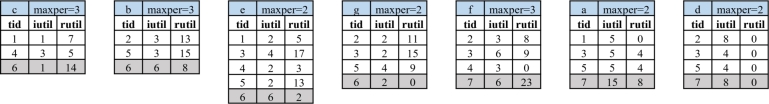
Incremental utility-lists of single items for the original database *D* and inserted set of transactions *N*.

Example 9 Consider the original database *D* and incremental database *N*, as depicted in Table 2. The order of items from the original database *D* is c≺b≺f≺a≺d≺e≺g. And the incremental utility-lists of database *D* are presented in Fig. 1. Additionally, considering both the original database *D* and the incremental transaction set *N*, we recalculate the TWU values for each item. The updated results are presented in Table 5. Consequently, the new order of the items becomes c≺b≺e≺g≺f≺a≺d. And the incremental utility-lists of database *D* and inserted set of transactions *N* is shown in Fig. 2.

With the insertion of the database *N*, it becomes necessary to reconstruct the incremental utility-lists. We utilize a temporary array [30] RuA[|D|] = 0 to assist in recalculating the rutil values, where |D| represents the number of transactions. Initially, the incremental utility-lists are sorted according to the order of the new items. According to Property 2, the iutil values remain unchanged, and thus there is no need to recompute the iutil values for the *ulD* part. For *ulN*, the corresponding iutil values are directly computed. To calculate the rutil values, we initialize all rutil values within the incremental utility-lists to 0. Then, we traverse the incremental utility-lists in reverse order, iterating through each (*tid*, iutil, rutil) tuple to perform the recalculations. We assign rutil=RuA[tid] and subsequently update RuA[tid]=RuA[tid]+iutil. This process is continued until all incremental utility-lists have been traversed, thus completing the reconstruction.

During our first database scan, we can calculate each item's maximum period, *maxper*. It is directly excluded if an item's maximum period exceeds the threshold *maxPer*. Suppose we define maxPer=2; then, in the original database *D*, items *c* and *b* are excluded. Similarly, items *c*, *b*, and *f* are excluded from the incremental database *DN*.

### 4.4 The IPHM algorithm

The IPHM Algorithm 1, designed for identifying periodic high-utility itemsets, encompasses several key steps across its 14 lines of pseudocode. Initially, global variables including a list of incremental utility-lists *LUL* and a set of promising single items $I^{⁎}$ are initialized (lines 1-2). The algorithm iterates twice over the original database *D*: the first to calculate the TWU and maximum period (*maxper*) for each item (line 3), and then filter all items based on the TWU threshold and maxPer threshold, inserting the promising items into $I^{⁎}$ (line 4). The second scan is used to construct incremental utility-lists for each promising item, which are then inserted into *LUL* and then build Estimated Utility Co-occurrence Structure (EUCS) (lines 5-6). After ordering the utility-lists by items' TWU values (line 7), the algorithm recursively processes the lists to identify PHUIs (line 8). The algorithm then adapts to incremental changes by scanning a set of new transactions *N*, recalculating TWU and *maxper* for each item, and updating $I^{⁎}$ and *LUL* accordingly (lines 9-11). This is followed by an update of *LUL* to recalculate the rutil (shown in Algorithm 2) (line 12). Then, a second scan of *N* to extend the incremental utility-lists and rebuild the EUCS according to full *iUL* (line 13), culminating in a final recursive processing step (line 14). This comprehensive approach allows for efficient identification of PHUIs in dynamically changing databases.

**Algorithm 1:**
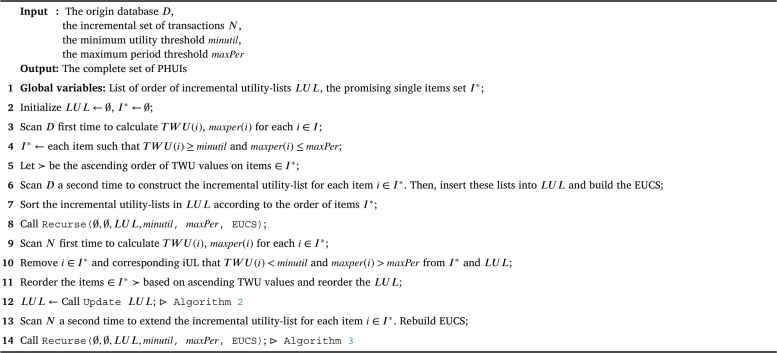
IPHM algorithm.

**Algorithm 2:**
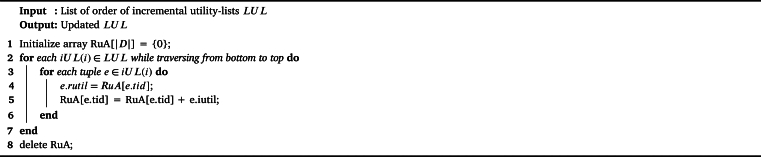
Update *LUL*.

As for the recursive search of PHUIs, the Algorithm 3 shows the detail of the recurse procedure. The process starts by iterating over each incremental utility-list iUL(Px) in iULs (line 1). When the total of iutil values in iUL(Px) is at least minutil and maxper is within maxPer (line 2), it outputs *Px* as a PHUI (line 3). Further, the algorithm examines whether the total of both iutil and rutil in iUL(Px) meets *minutil* and *maxper* is within *maxPer* (line 5); if so, it initializes an empty set for the extensions of *Px* incremental utility-lists (line 6), iterating over each iUL(Py) where *y* succeeds *x* (line 7). For valid (x,y) pairs in EUCS with counts exceeding *minutil* (line 8), it forms *Pxy* as the union of *Px* and *Py* (line 9), calling constructs iUL(Pxy) (shown in Algorithm 4) (line 10), and adds this to the extensions set (line 11). The algorithm recurses with updated parameters (line 14), looping through each element (lines 1-16) to efficiently identify all PHUIs.

**Algorithm 3:**
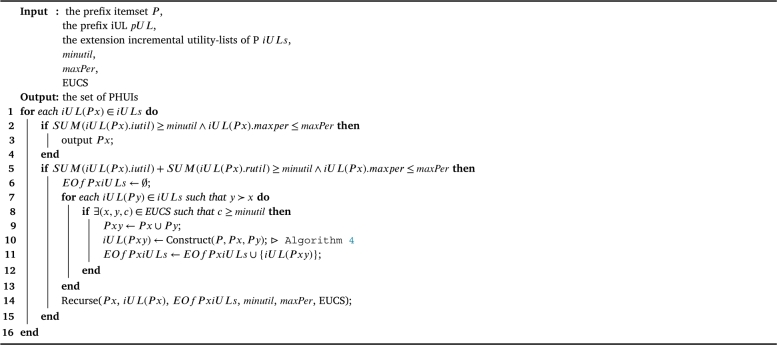
Recurse.

**Algorithm 4:**
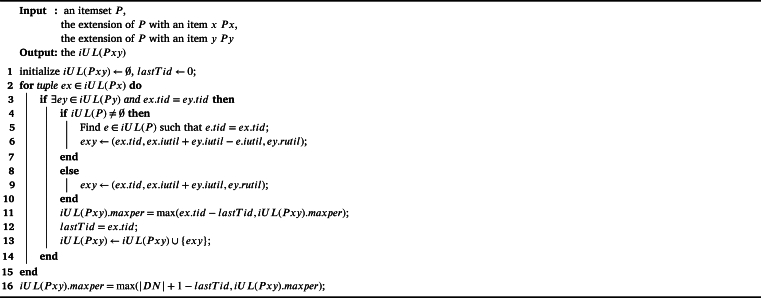
Construct *iUL*.

Example 10 To better elucidate the proposed IPHM algorithm, we use the database presented in Table 2 and the unit profit table presented in Table 3 as illustrative examples. Suppose that *minutil* is 23 and *maxPer* is 2.

**Figure 3:**
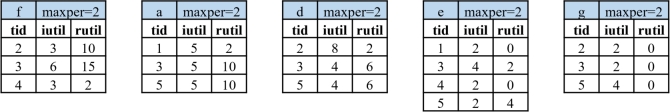
*LUL* for the original database *D*.

For the original Database *D*:

1) IPHM firstly scans the original database *D*, obtaining the TWU values as presented in Table 4 and the *maxper* values for all individual items. After applying the TWU and periodic pruning strategy and sorting by TWU values, we find that $I^{⁎}=\{f,a,d,e,g\}$.
2) In its second scan of the database, the algorithm constructs incremental utility-lists (*iUL*) for the individual items within $I^{⁎}$. These iULs are then inserted into *LUL* based on the order of items, as depicted in Fig. 3. Then, we build the EUCS shown in Table 7 for the items within $I^{⁎}$.

**Table 7:** The EUCS for the items within *I*^⁎^ in *D*.

	*f*	*a*	*d*	*e*	*g*
*f*		21	34	26	34
*a*			36	28	36
*d*				36	49
*e*					36
*g*
3) The recursive procedure commences its operation starting from iUL(f), where the sum of its iutil values is 12, which is less than the *minutil* threshold, hence not qualifying as a PHUI (Periodic High-Utility Itemset). However, the sum of both iutil and rutil values for iUL(f) is 39, which is not less than *minutil*. Therefore, it is feasible to extend *f*. For the subsequent items {a, d, e, g}, the value within EUCS for the item pair f,a is 21, which is less than *minutil*. Consequently, *iUL* for the itemset {*f*, *a*} is not constructed and is not included in the extension set EOfPxiULs. For the item pairs (f,d), (f,e), and (f,g), we construct the corresponding itemsets {f,d},{f,e},{f,g} and their incremental utility-lists (*iUL*). These are then added to the extension set EOfPxiULs. Then, the recursive procedure continually starts from the itemset {f,d} and it's corresponding iUL({f,d})={(2,11,2),(3,10,6)},maxper=3. The incremental utility-list iUL({f,d}) does not satisfy the conditions of the two ‘if’ statements in Algorithm 3, resulting in the termination of recursion for the itemset {f,d}. Similarly, the itemsets {f,e} and {f,g} also fail to pass the maximum period pruning strategy, leading to the conclusion of their respective recursive processes. The recursive procedure for item *f* concludes, and the algorithm then recursively processes the item *a*. Ultimately, the itemset {a,e} is identified as a Periodic High-Utility Itemset (PHUI). In summary, subsequent recursion also reveals the itemset {d,g} as a PHUI. Thus, the final set of PHUIs mined from the database *D* is {{a,e},{d,g}}.
4) Next, lines 9-14 of the main program incorporate the incremental database, the non-empty transaction set *N*. These steps involve recalculating the TWU and *maxper* information for items in $I^{⁎}$, resulting in an updated $I^{⁎}$ set as {e, g, a, d}. Additionally, the order of *LUL* is updated accordingly. The Update *LUL* algorithm is executed to recalculate the rutil values. Subsequently, the second scan of the transaction set *N* is performed, during which the iULs in *LUL* undergo a merge operation, incorporating the new transaction information into each *iUL*. The new *LUL* is shown in Fig. 4. This process is followed by the reconstruction of the EUCS shown in Table 8. Finally, the recursive procedure is executed to mine the PHUIs ={{a},{d}}.

**Figure 4:**
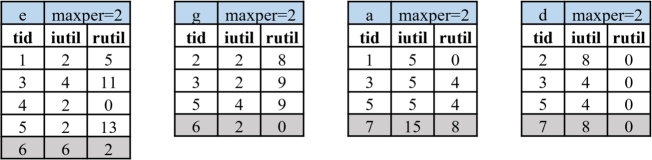
*LUL* for the database *D* and *N*.

**Table 8:** The EUCS for the items within *I*^⁎^ in *DN*.

	*e*	*g*	*a*	*d*
*e*		38	37	30
*g*			30	40
*a*				53
*d*
5) Triggered by adding a new transaction set, the steps corresponding to step 4, specifically lines 9-14 of the main program, are repeated. This process enables the mining of PHUIs from the updated database.

## 5 Experimental evaluation

This section presents a series of experiments designed to assess the effectiveness of the IPHM algorithm. The algorithm is compared with PHM [6], which is only operable in the static database environment. Therefore, the database is partitioned into batches, and PHM is executed in a batch processing mode.

### 5.1 Experiment setup

The experiments were conducted on a computer with a 12th-generation Core i5 processor, 16 GB of available memory, and a Windows 11 operating system. We implemented all the algorithms in Java. Experiments were carried out on real-world databases, each possessing distinct characteristics. Table 9 presents the characteristics of these datasets, where T, I, and A, respectively, represent the number of transactions, the number of distinct items, and the average length of transactions. The Foodmart and Chainstore databases are a real-world dataset with authentic utility and quantity information. The Retail, Connect, Pumsb and Kosarak databases are real-world datasets. However, their utility and quantity information are synthetic. All the databases are available for download at http://www.philippe-fournier-viger.com/spmf.

**Table 9:** Characteristics of datasets.

Dataset	T	I	A
Retail	88162	16470	10.30
Connect	67557	129	43
Pumsb	49046	2113	74
Foodmart	4141	1559	4.42
Kosarak	990,002	41270	8.1
Chainstore	1,112,949	46086	7.23

Modifications were made to the PHM algorithm for mining item sets that satisfy the proposed maximum period threshold and the minimum utility threshold. Additionally, its operational mode was modified to batch processing to simulate the form of an incremental database.

### 5.2 Influence of the minutil threshold

The initial evaluation focused on assessing the impact on performance under different values of *minutil* at a fixed insertion ratio. Due to the distinct characteristics of different databases, varied insertion ratios were set to more effectively evaluate the algorithm's performance. For the FoodMart database, an insertion ratio of 5% was set. For Connect, Retail and Chainstore, the insertion ratio was set at 10%, while for Pumsb, it was set at 1%. For Kosarak, the insertion ratio was set at 2%. For instance, consider a database containing 100k transactions. With an insertion ratio of 1%, the process starts from 1,000 transactions (100k × 1%) and increments by 1,000 transactions each time until the entire database is added. In the batch processing mode of the PHM algorithm, the first iteration involves mining the first 1,000 transactions; the second iteration mines the first 2,000 transactions, and so on, cumulatively calculating the total duration. In contrast, the proposed IPHM algorithm directly scans the newly inserted segment without the need to re-traverse the entire database.

**Figure 5:**
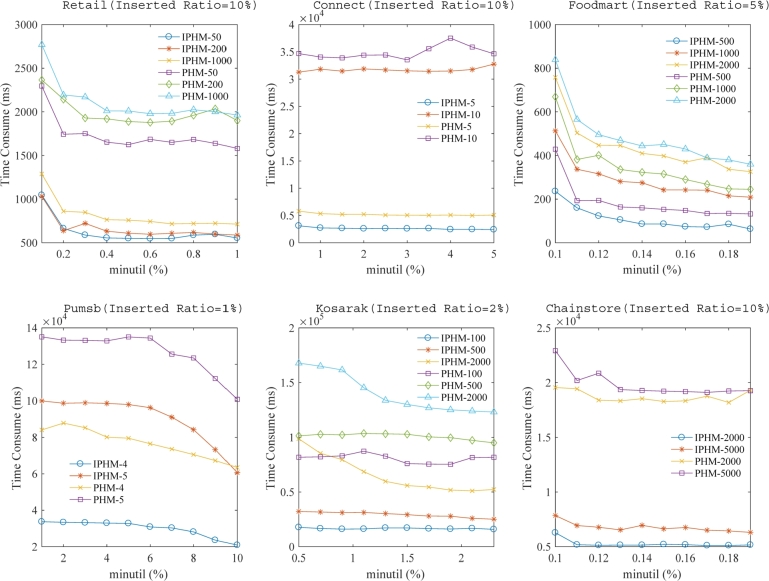
A comparison of the cumulative run times at various *minutil* thresholds under a fixed inserted ratio.

We adopted the approach of taking a percentage of the utility in the already inserted portion of the database to determine specific *minutil* values. This led to the generation of runtime comparisons as illustrated in Fig. 5. For the same database, experiments were conducted with several maximum period thresholds (*maxPer*) to meticulously evaluate the efficacy of both the IPHM and PHM algorithms. In sub-figure (a), under the context of the Retail database with an Inserted Ratio set to 10%, the line labeled “IPHM-50” signifies that *maxPer* is set to 50. The vertical axis, labeled as “Time Consume”, represents the cumulative run time required for the complete insertion of the entire database.

In Fig. 5, we observe that across each distinct dataset, when operating with the same insertion ratio while varying the *minutil* values, the proposed IPHM algorithm consistently demonstrates a lead in cumulative time over the PHM algorithm to varying extents. Specifically, on the Retail dataset, we set three different *maxPer* values: 50, 200, and 1000, for the comparative analysis of the two algorithms. The analysis showed a decrease in runtime as the *minutil* value rises and an extension in duration with a higher *maxPer* value. In comparing the run times of the IPHM and PHM algorithms, our analysis revealed that, under the same *minutil* and *maxPer* values, the IPHM algorithm consistently delivers better performance in runtime compared to the PHM algorithm. Moreover, on the Connect, Foodmart, Pumsb, Kosarak and Chainstore datasets, the IPHM algorithm consistently demonstrates shorter run times than the PHM algorithm under the same threshold constraints. Notably, when examining the Foodmart dataset, the difference in time consumption between the two algorithms is not as pronounced. This phenomenon is linked to the sparse characteristics of the Foodmart dataset, where the advantage of IPHM in inheriting the capability to mine the dataset is less apparent in sparse datasets. As previously mentioned, the PHM algorithm operates in a batch processing mode. Consequently, whenever new transactions are added to the original database, PHM necessitates a complete rescan of the database. This entails the PHM algorithm rebuilding its required data structures and recommencing the mining operation from scratch for the incremental database, leading to significant inefficiencies. Conversely, IPHM adeptly updates an incremental utility-list for individual items and navigates solely through the newly added transactions. In summary, our approach, especially when adjusting the *minutil* value, shows a marked decrease in runtime relative to the PHM algorithm on the Retail, Connect, Foodmart, Pumsb, Kosarak and Chainstore datasets, with factors of improvement being 2.77, 1.17, 1.22, 1.7, 2.88 and 3.2 respectively.

Table 10 compares the average maximum memory consumption under varying *minutil* values in the experiment. The lowest values are highlighted in bold. All memory metrics were obtained through the use of the standard Java API. It is observed that for smaller to medium-sized datasets and under various maxPermaxPer thresholds, the memory usage of our proposed algorithm generally remains less than that of the PHM algorithm. However, as the dataset size increases to larger scales, this memory advantage becomes less pronounced, and in some cases, our algorithm may require comparable or slightly more memory than PHM.

**Table 10:** Average maximum memory usage across various *minutil* values.

Dataset	maxPer	IPHM	PHM
Retail	50	**227.2**	288.9
	200	**224.1**	272.4
	1000	**270.2**	279.1

Connect	5	**258.8**	306.5
	10	**372.4**	495.5

Foodmart	500	**94.6**	149.2
	1000	**137.3**	149
	2000	**146.4**	148.9

Pumsb	4	**357.5**	453.4
	5	**374.2**	462.6

Kosarak	100	2869.5	**2074.3**
	500	2936.6	**2099**
	2000	2696.7	**2206.3**

Chainstore	2000	1229	**591.2**
	5000	1289.9	**693**

### 5.3 Influence of the insertion ratio

In this subsection, we explore the performance of algorithms under different inserted ratios while keeping the *minutil* threshold constant. For the datasets Retail, Connect, Foodmart, Pumsb, Kosarak and Chainstore we set the *minutil* proportions to 0.1%, 5%, 0.1%, 1%, 1% and 0.1% respectively. The aim is to assess and compare the efficiency of different algorithms by systematically increasing the inserted ratio and analyzing the impact on their performance.

**Figure 6:**
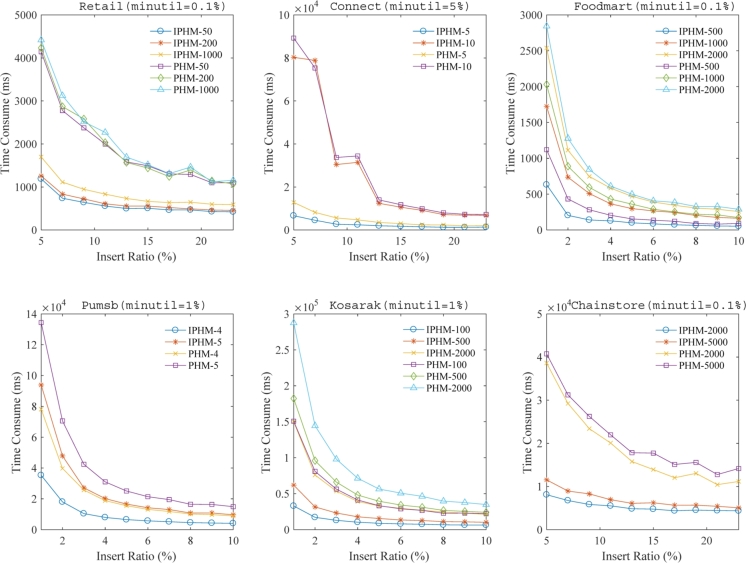
A comparison of the cumulative run times at various inserted ratios under a fixed *minutil* threshold.

Fig. 6 presents a detailed comparison of cumulative run times. In this figure, it becomes apparent that the proposed IPHM algorithm consistently surpasses the PHM algorithm in terms of run time under the same *maxPer* and *minutil* thresholds across various inserted ratios on the datasets Retail, Foodmart, Pumsb, Kosarak and Chainstore. In the case of the Connect dataset, it is noteworthy that at various inserted ratios, the run time for the IPHM algorithm is predominantly less compared to that of the PHM algorithm. This demonstrates the overall efficiency advantage of IPHM in different inserted ratio scenarios. Additionally, it's noted that with a diminishing inserted ratio, the runtime required by both the IPHM and PHM algorithms escalates. However, the pace at which runtime extends for the IPHM algorithm is markedly more gentle than that observed for the PHM algorithm. This implies that in scenarios with frequent new transaction insertions (i.e., lower inserted ratios), the efficiency advantage of the IPHM algorithm becomes increasingly pronounced. In summary, when varying the inserted ratio, our method shows a substantial decrease in runtime relative to the PHM algorithm on the Retail, Connect, Foodmart, Pumsb, Kosarak and Chainstore datasets, with improvement factors being 2.85, 1.13, 1.21, 1.7, 2.4 and 3.25 respectively.

For memory consumption, Table 11 lists the average maximum memory consumption of the IPHM and PHM algorithms under different inserted ratios. The minimum values are highlighted in bold. We can observe that the memory usage of our proposed algorithm varies in comparison to the PHM algorithm across different databases and various maxPermaxPer threshold values. For smaller to medium-sized datasets, our algorithm generally demonstrates lower memory consumption than PHM. However, as the database size increases to larger scales, the memory usage patterns become more complex, with our algorithm sometimes requiring comparable or slightly more memory than PHM in certain scenarios.

**Table 11:** Average maximum memory usage across various inserted ratios.

Dataset	maxPer	IPHM	PHM
Retail	50	**197.1**	273.6
	200	**264.9**	281
	1000	**233.3**	265.7

Connect	5	**307.1**	350.6
	10	**386.9**	584.3

Foodmart	500	**120.6**	150.1
	1000	**140.7**	150.3
	2000	**151**	151.9

Pumsb	4	**345.4**	376.9
	5	**345.3**	386.8

Kosarak	100	2642.3	**1998.6**
	500	2811.1	**1965.5**
	2000	2867.1	**2076.7**

Chainstore	2000	1194.1	**575**
	5000	2047.9	**658.8**

### 5.4 Scalability evaluation

In the last experiment, we conducted scalability tests to examine whether the time consumption and memory usage of our proposed IPHM algorithm demonstrate linear growth. This assessment aimed to establish the scalability of our algorithm under increasing data volumes and complexity.

**Figure 7:**
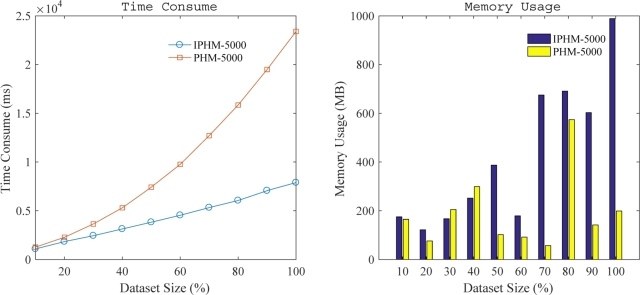
Scalability test in Chainstore dataset.

Scalability tests were carried out on the Chainstore dataset, setting the *minutil* threshold at 0.1%, the *maxPer* threshold at 5000, and the Inserted Ratio at 10%. Fig. 7 depicts the run time and memory usage under varying dataset sizes. It was observed that the runtime of both the IPHM and PHM algorithms increases as the dataset size expands, with IPHM demonstrating a more favorable scaling pattern. Specifically, IPHM exhibits a near-linear growth in runtime, while PHM shows a steeper increase, especially for larger datasets.

Regarding memory consumption, both algorithms show fluctuations as the dataset size increases. The IPHM algorithm tends to use more memory than PHM in most cases, particularly for larger datasets. However, it's worth noting that the memory usage patterns for both algorithms are not strictly monotonic and show some variability across different dataset sizes.

These observations suggest that while the IPHM algorithm offers improved runtime scalability compared to PHM, its memory efficiency presents a more complex picture. The IPHM algorithm demonstrates a trade-off, achieving better time performance at the cost of generally higher memory usage. This trade-off becomes more pronounced as the dataset size increases.

In conclusion, the IPHM algorithm exhibits good scalability in terms of runtime for handling large datasets, but careful consideration should be given to memory requirements when dealing with very large datasets.

## 6 Conclusion

In this paper, we introduce a new approach, named IPHM, aimed at effectively mining periodic high-utility itemsets in incremental databases. Addressing the challenges posed by incremental database environments, we developed a new algorithm that incorporates various methods and structures. Firstly, an incremental utility-list structure is proposed to retain essential information on single items or itemsets. This structure enables the rapid construction and maintenance of incremental utility-lists for single items without the need to rescan the entire database. Secondly, we introduced a pruning strategy that eliminates single items not meeting the *maxPer* threshold during the initial database scan, thus avoiding the construction of their utility lists. Furthermore, a method for maintaining a sorted list of utility-lists is proposed, ensuring their effectiveness in an incremental data environment. Finally, various experiments were conducted, comparing our method with existing approaches. The results demonstrate the effectiveness and efficiency of our proposed algorithm. In future work, we aim to delve into transaction weight information. Specifically, we will investigate the concept of assigning lower weights to past information in a continuously increasing database. This approach will redefine the significance of older transactions relative to more recent ones, potentially offering a more dynamic and contextually relevant data analysis in incremental databases.

## CRediT authorship contribution statement

**Huiwu Huang:** Writing – review & editing, Writing – original draft. **Shixi Chen:** Writing – review & editing, Software, Formal analysis. **Jiahui Chen:** Writing – review & editing, Supervision, Methodology, Conceptualization.

## Declaration of Competing Interest

The authors declare that they have no known competing financial interests or personal relationships that could have appeared to influence the work reported in this paper.

## Data Availability

No data are associated with this article.
